# Oxidants and Antioxidants in the Redox Biochemistry of Human Red Blood Cells

**DOI:** 10.1021/acsomega.2c06768

**Published:** 2022-12-28

**Authors:** Matias N. Möller, Florencia Orrico, Sebastián F. Villar, Ana C. López, Nicolás Silva, Marcel Donzé, Leonor Thomson, Ana Denicola

**Affiliations:** † Laboratorio de Fisicoquímica Biológica, Instituto de Química Biológica, Facultad de Ciencias, Universidad de la República, Montevideo 11400, Uruguay; ‡ Centro de Investigaciones Biomédicas (CEINBIO), Universidad de la República, Montevideo 11800, Uruguay; § Laboratorio de Enzimología, Instituto de Química Biológica, Facultad de Ciencias, Universidad de la República, Montevideo 11400, Uruguay; ∥ Departamento de Medicina Transfusional, Hospital de Clínicas, Facultad de Medicina, Universidad de la República, Montevideo 11600, Uruguay

## Abstract

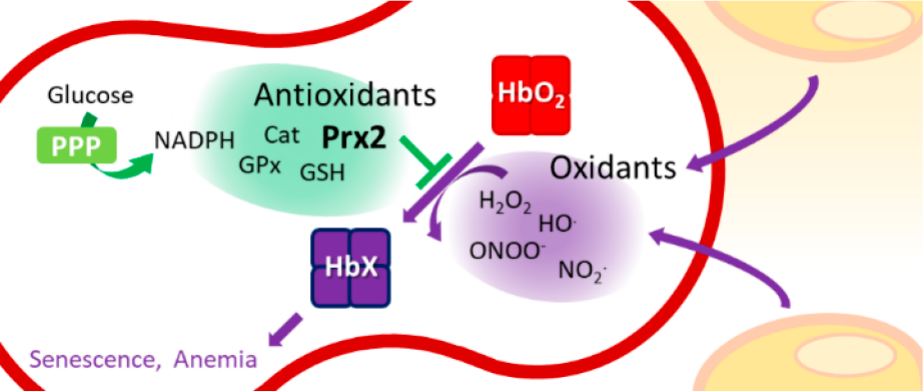

Red blood cells (RBCs) are exposed to both external and internal sources of oxidants that challenge their integrity and compromise their physiological function and supply of oxygen to tissues. Autoxidation of oxyhemoglobin is the main source of endogenous RBC oxidant production, yielding superoxide radical and then hydrogen peroxide. In addition, potent oxidants from other blood cells and the surrounding endothelium can reach the RBCs. Abundant and efficient enzymatic systems and low molecular weight antioxidants prevent most of the damage to the RBCs and also position the RBCs as a sink of vascular oxidants that allow the body to maintain a healthy circulatory system. Among the antioxidant enzymes, the thiol-dependent peroxidase peroxiredoxin 2, highly abundant in RBCs, is essential to keep the redox balance. A great part of the RBC antioxidant activity is supported by an active glucose metabolism that provides reducing power in the form of NADPH via the pentose phosphate pathway. There are several RBC defects and situations that generate oxidative stress conditions where the defense mechanisms are overwhelmed, and these include glucose-6-phosphate dehydrogenase deficiencies (favism), hemoglobinopathies like sickle cell disease and thalassemia, as well as packed RBCs for transfusion that suffer from storage lesions. These oxidative stress-associated pathologies of the RBCs underline the relevance of redox balance in these anucleated cells that lack a mechanism of DNA-inducible antioxidant response and rely on a complex and robust network of antioxidant systems.

## 1 Introduction

Red blood cells (RBCs) are the most abundant cells in the blood. The average hematocrit of 40–45% correlates with a red blood cell count of 4.8–5.4 × 10^12^ cells per L, approximately 1000 times more than white blood cells and 20 times more than platelets. Their principal function is to transport oxygen to the tissues. To accomplish this function, RBCs have high intracellular concentration of hemoglobin (Hb), and binding of oxygen to Hb is highly regulated. Nevertheless, a side effect is the generation of superoxide radicals from autoxidation of Hb that must be controlled. In addition, reactive species produced in the vasculature (endothelium and other blood cells) can diffuse and reach the RBCs. To cope with this, RBCs are equipped with a battery of antioxidants, low molecular weight like glutathione (GSH), and enzymes like peroxiredoxin 2, which are described in detail below. The efficient decomposition of oxidant species along with repair mechanisms, the elimination through proteasomal degradation of altered proteins, and vesiculation of irreversibly damaged cellular structures^1^ keep the RBCs functional for 120 days in circulation. The redox status of the RBC is important not only to keep an adequate supply of oxygen to every tissue cell but also to keep a healthy circulatory system due to RBC interactions with other blood cells and the vascular endothelium.

RBCs lack organelles like the nucleus and mitochondria; thus, no new biosynthesis of proteins takes place in the mature erythrocyte, and energy relies on glycolysis. Glucose is the source of energy as ATP and also the source of reducing equivalents such as NADH (glycolysis) and NADPH (pentose phosphate pathway). ATP is mainly tasked with maintaining transmembrane ion gradients, membrane integrity, and the interaction with the cytoskeleton. This is crucial to maintain the human RBC biconcave shape that gives RBCs flexibility to circulate into capillaries as well as to prevent hemolysis that will release Hb into the intravascular space with deleterious consequences.

In this review we focus on the redox metabolism of the human RBC, describing oxidants and antioxidants involved in the maintenance of redox balance. Relevant pathologies associated with RBC oxidative stress like sickle cell disease and thalassemia are also described.

## 2 The Human RBC

Human RBCs are shaped as biconcave disks with 8 μm maximal diameter, 2 μm thickness, approximately 90 fL in volume, and 140 μm^2^ surface area.^2,3^ The biconcave shape results in a high surface area to volume ratio that not only favors gas exchange but also is essential for RBC deformation. Their particular shape and deformability allow RBCs to pass through narrow capillaries and the interstitial slits in the spleen.^3^ The ability of RBCs to deform depends on the properties and interactions of the RBC cytoskeleton, membrane components (proteins and lipids), and the cellular hydration state and viscosity.^4^ The plasma membrane of RBCs is composed of 43% lipids, 49% proteins, and 8% carbohydrates in mass.^5^ The lipid fraction is composed of cholesterol (45%, mol/mol) and phospholipids (55%, mol/mol). The most abundant phospholipids are phosphatidylethanolamine (PE), phosphatidylcholine (PC), and sphingomyelin (SM), with lower amounts of phosphatidylserine (PS), phosphatidylinositol (PI), and glycolipids.^5,6^ The lipids are distributed asymmetrically across the membrane, with negatively charged phospholipids (PS and PI) facing the cytosol and glycolipids facing the exterior. The exposure of PS in the outer face of the membrane is recognized by macrophages as a senescence signal, and these RBCs are subsequently phagocytosed and removed from circulation.^7^

The interaction between the cytoskeleton and the membrane proteins involves a large number of transmembrane proteins. The cytoskeleton gives RBCs their ability to deform and return to shape^4^ and is composed of long α- and β-spectrin heterotetramers that form coiled fibers anchored to the membrane by multiprotein nodal points that are bound to band 3^8^. The most abundant transmembrane protein is the band 3 anion transport protein (SLC4A1), with over 10^6^ copies per cell. It is an anion channel responsible for the bicarbonate/chloride exchange.^9^ Other transporter proteins are also abundant, such as the glucose transporter Glut1 (1.7 × 10^5^ per cell) and aquaporin 1 (6 × 10^4^ per cell), involved in water transport in response to osmotic gradients.^9^ ATPases in the membrane are less abundant but are responsible for maintaining ion gradients, and particularly relevant are the Na^+^/K^+^-ATPase and Ca^2+^-ATPase.^9^ Not as abundant but important in the membrane dynamics of RBCs is the mechano-sensitive PIEZO 1 calcium channel.^10^

The cytosol in RBCs is largely dominated by Hb (20 mM subunit concentration), followed by carbonic anhydrase (300 μM), Prx2 (250 μM), and then proteins involved in oxidant detoxification and the metabolism of glucose.^9,11^ Hb is a heterotetramer composed of two α-globin and two non-α-globin chains with one heme molecule bound to each globin. The α-globin locus, located on chromosome 16, contains the α1 and α2 genes, and the β-globin locus, on chromosome 11, contains the β, δ, and γ loci. According to the developmental stage, the major Hb tetramer changes from α2γ2 (HbF) in the fetal period to α2β2 (HbA) in adulthood. In mature RBCs, HbA is the major component, reaching a 5 mM concentration (20 mM heme). The redox state of the heme is very important since oxygen binds to the ferrous heme (Fe^II^), forming oxyhemoglobin (oxyHb). The ferric form (Fe^III^) or methemoglobin (metHb) is unable to bind oxygen. MetHb forms spontaneously at a rate of 3% Hb per day, and its formation can be accelerated by drugs such as benzocaine and chemicals such as nitrite and can also have genetic causes.^12^ The RBC contains a metHb reductase enzyme system, which includes cytochrome *b*_5_ reductase and cytochrome *b*_5_, to catalyze the reduction of the heme iron at the expense of NADH.^13^

## 3 Reactive Species Derived from RBCs and Other Sources in the Vasculature

RBCs are exposed to both endogenous and exogenous reactive species. These reactive species, usually referred to in the literature as reactive oxygen species (ROS) or reactive nitrogen species (RNS), are small molecules, oxidants in general, some of which are radicals. It should be emphasized that ROS are not one chemical oxidant species but a wide group of molecules. We will briefly describe the main reactive species involved in oxidative damage to RBCs.

### Superoxide

Superoxide (O_2_^•–^) is the one-electron reduction product of oxygen and can be produced in RBCs by oxyHb autoxidation that also leads to the production of metHb (Hb(Fe^III^)) (eq 1).
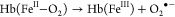
1Autoxidation occurs at a slow rate (*k* = 4.5 × 10^–7^ s^–1^)^14^ and is accelerated in partially deoxygenated Hb.^15^ The reverse reaction (reduction of metHb by O_2_^•–^) is also possible with *k* = 4000 M^–1^ s^–1^.^16^ Furthermore, O_2_^•–^ can be produced enzymatically by NADPH oxidases (Nox2, EC 1.6.3.1) or as a side product of mitochondrial respiration in endothelial cells and leukocytes.^17^ Also, the enzyme xanthine oxidase (EC 1.17.3.2) that binds to glycosaminoglycans in endothelial surfaces can also produce O_2_^•–^.^17^ Although O_2_^•–^ can dismutate spontaneously to oxygen and hydrogen peroxide (H_2_O_2_) (eq 2), the enzyme superoxide dismutase (SOD1, EC 1.15.1.1), present in RBCs, accelerate this reaction several fold (see below).^18^
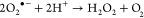
2Superoxide per se is not very damaging and is not a strong oxidant; even more, it can act as a reductant sometimes. Reaction with lipid hydroperoxides has been proposed to yield alkoxyl radicals and promote lipid oxidation, eventually leading to RBC lysis,^19^ but the concentration of lipid hydroperoxides in fresh RBCs is very low. An alternative toxic pathway is the reaction with nitric oxide (NO^•^) to yield peroxynitrite (see below).

### Hydrogen Peroxide

**Figure 1:**
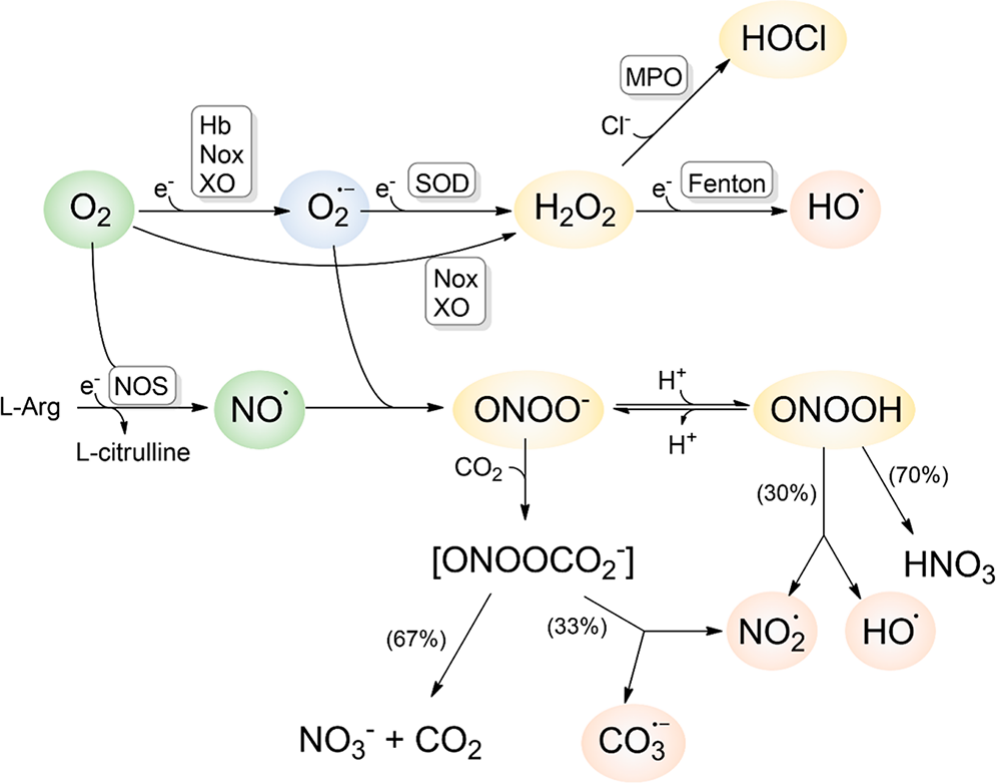
Reactive species produced in the vascular system relevant to RBCs. Oxygen and NO^•^ are the two main ingredients required for the generation of reactive species that will lead to biomolecular damage. Details about each pathway are given in the text.

Most H_2_O_2_ derives from O_2_^•–^, but some oxidases can yield H_2_O_2_ directly (Figure 1).^17^ In addition to its microbicidal properties, H_2_O_2_ modulates different cell functions including endothelial cell proliferation and survival, platelet recruitment, insulin secretion, and cardiac remodeling induced by hypertension.^20−25^ H_2_O_2_ can diffuse across the RBC membrane very rapidly, without involving aquaporins.^26^ H_2_O_2_ is not a very oxidizing molecule but can yield the highly reactive hydroxyl radical (HO^•^) by reduction by metals (Fenton reaction) (eq 3) (Figure 1).
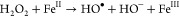
3Under normal physiological conditions, the concentration of free or labile iron available for the Fenton reaction is kept very low by extracellular and intracellular proteins, preventing the deleterious reactions mediated by iron.^27^ However, different genetic disorders can led to iron over load.^28^ Such is the case of the ferrireductase Steap3, which reduces Fe^III^ to Fe^II^ after DMT1 trans membrane transport.^29^ Mice lacking Steap3 are deficient in erythroid ferrireductase activity and suffer from an iron deficiency anemia.^30^ On the other hand, increased levels of Steap3 result in degradation of cellular membranes through lipid peroxidation, leading to a failure of RBC homeostasis and hemolysis/clearance of RBCs,^31^ likely because of Fenton-type reactions due to increased Fe^II^.

Two thirds of body iron is present in circulating RBCs as part of Hb.^32^ In the RBCs, the reaction of H_2_O_2_ with the heme of oxyHb yields the pro-oxidizing ferrylHb as an intermediate (eq 4), which can react with a second molecule of H_2_O_2_ to yield metHb (eq 5). The reaction of H_2_O_2_ with metHb yields ferrylHb with a protein radical that is readily detected by EPR (eq 6).^33^
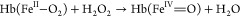
4
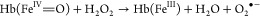
5
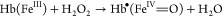
6To prevent this potentially harmful reactions, the RBCs are equipped with a robust system to reduce H_2_O_2_, including peroxiredoxin 2, catalase, and glutathione peroxidase, which will be discussed in detail below.^34^

### Hydroxyl Radical

One of the most oxidizing radicals in biology is HO^•^.^35^ It can react with most biomolecules at diffusion-controlled rates, leading to protein, DNA, and lipid damage.^36^ It can be formed from the reduction of H_2_O_2_, mainly by reduced metal atoms, such as Cu^+^ and Fe^2+^ (Fenton reaction) (eq 3), and also from peroxynitrite homolysis (see below, Figure 1). In some conditions, excess labile iron in RBCs can contribute to oxidative damage by this mechanism (as in sickle cell disease, see below). Because of the extremely high rates of reaction of HO^•^ with biomolecules, the best mechanism of defense in cells is to prevent its formation by consuming H_2_O_2_ very rapidly (see below).

### Nitric Oxide

Despite being a radical molecule, NO^•^ is formed in vivo as the product of specific enzymes called nitric oxide synthases, (NOS, EC 1.14.13.39).^37^ NO^•^ is an autocrine and paracrine signaling molecule which promotes vascular relaxation, inhibits platelet aggregation, decreases inflammation, and modulates the neural activity.^38^ NOSs are complex enzymes that are constitutively expressed in endothelial cells (NOS3) and can be induced in leukocytes (NOS2).^37^ Recently an endogenous RBC NOS3 has been reported in low abundance and with functions still poorly understood.^39^ These enzymes catalyze the conversion of l-arginine to NO^•^ and l-citrulline. The constitutive endothelial NOS3 responds to changes in Ca^2+^ concentration to increase the production of NO^•^. NO^•^ produced by endothelial cells can diffuse through cell membranes virtually unhindered and reach underlying smooth muscle cells to cause relaxation through the activation of soluble guanylate cyclase.^40,41^ A large part of NO^•^ will diffuse to the lumen of blood vessels, reacting mostly with oxyHb in RBCs to yield nitrate and metHb (*k* = 8.9 × 10^7^ M^–1^ s^–1^, eq 7).^42,43^ The exact amount of NO^•^ that will react with oxyHb depends mainly on the number of RBCs and the size of the RBC-free layer created near the vessel wall by the blood flow. The diffusion across this RBC-free layer will be the main barrier to NO consumption by RBCs.^44^
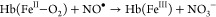
7Another possible destiny of NO^•^ is the reaction with O_2_^•–^ to yield the highly oxidizing peroxynitrite (ONOO^–^) (Figure 1).

### Peroxynitrite

The reaction between the two radicals, O_2_^•–^ and NO^•^, occurs at diffusion-controlled rates to yield the peroxynitrite anion (ONOO^–^) (*k* = 4–16 × 10^9^ M^–1^ s^–1^; eq 8) (Figure 1).^45^
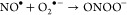
8Peroxynitrite is a powerful one- and two-electron oxidant.^45^ With a p*K*_a_ of 6.8, at physiological pH peroxynitrite will be a mixture of ONOO^–^ and the protonated peroxynitrous acid (ONOOH). ONOOH can decay relatively slowly to nitric acid plus a 30% fraction of HO^•^ and nitrogen dioxide (NO_2_^•^) (eq 9) (Figure 1).
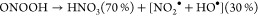
9In RBCs, the main targets of peroxynitrite would be Prx2 (*k* = 1.4 × 10^7^ M^–1^ s^–1^, pH 7.4 and 25 °C), oxyHb (*k* = 5.8 × 10^4^ M^–1^ s^–1^), and CO_2_ (*k* = 5.8 × 10^4^ M^–1^ s^–1^).^46−49^ Prx2 would detoxify peroxynitrite, but if Prx2 is oxidized the reaction with oxyHb occurs very rapidly with isomerization of peroxynitrite to nitrate, some production of superoxide anions, and finally oxidation of Hb to metHb^47^ (eq 10).
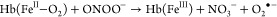
10The reaction with CO_2_ yields the secondary radicals NO_2_^•^ and the carbonate radical (CO_3_^•–^) in 33% yield (eq 11) (Figure 1).^45^
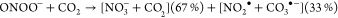
11The carbonate radical is very reactive and will react rapidly with sulfur-containing molecules, aromatic residues in proteins, ascorbate, and urate (>10^7^; 10^7^–10^9^; 10^9^; 10^8^ M^–1^ s^–1^, respectively).^50^

### Nitrogen Dioxide

Sources of NO_2_^•^ include peroxynitrite homolytic decay^46^ (Figure 1), the environment, as NO_2_^•^ is one of the main components of smoke and smog,^51^ the autoxidation of NO^•^ in lipid membranes,^52^ and the oxidation of nitrite by heme peroxidases.^53^ NO_2_^•^ is a radical, and it is very reactive to a variety of biomolecules, including thiolate-containing molecules, ascorbate, and urate (*k* ∼ 10^8^; 1.8–3.5 × 10^7^; 2 × 10^7^ M^–1^ s^–1^, respectively^50^), and it can also initiate lipid peroxidation.^54^ In RBCs, it is likely that both Hb thiols and GSH are the main targets of NO_2_^•^, whereas urate would be the main target in plasma.

### Hypochlorous Acid

The leukocytes, neutrophils, and monocytes are recruited to sites of infection where they phagocytize and kill invading pathogens. Upon infection-related or inflammatory stimuli, these leukocytes generate large amounts of H_2_O_2_ via Nox2 and release myeloperoxidase (MPO).^55^ MPO is a hemeprotein that uses H_2_O_2_ to oxidize chloride to hypochlorous acid (HOCl) (Figure 1), which is highly cytotoxic.^55^ HOCl participates in both oxidation and chlorination reactions. Chlorination reactions are evident by the formation of chloramines and 3-chlorotyrosine. Reaction with sulfur-containing residues such as cysteine and methionine are expected to be the most important reactions in biology because of their abundance and reactivity (at pH 7.4, k = 3.6 × 10^8^ and 3.4 × 10^7^ M^–1^ s^–1^, respectively).^56^ HOCl and some chloramines can diffuse across the RBC membrane and react preferentially with cytosolic thiols.^57^ Unlike H_2_O_2_ and peroxynitrite, HOCl does not prefer Prx2 but will react with GSH and likely Hb thiols.^57^

### Hydrogen Sulfide

H_2_S is not an oxidant but is a relevant redox reactive species with biological effects, including vasorelaxation, neurotransmission, and pro- or anti-inflammatory effects, depending on the pathology.^58^ Both endothelial cells and RBCs have enzymes that synthesize H_2_S, though unequally distributed. Endothelial cells produce H_2_S mainly through cystathionine γ-lyase (CSE) and mercaptopyruvate sulfur transferase (MST), whereas RBCs produce H_2_S via MST.^59,60^ H_2_S is slightly hydrophobic and can diffuse freely unhindered by plasma membranes, indicating that RBCs will also be exposed to H_2_S produced by the endothelium.^61,62^ H_2_S reacts with metHb in RBCs at moderate rates (*k*_on_ = 3.2 × 10^3^ M^–1^ s^–1^, eq 12) to form an intermediate iron-bound sulfidated Hb, which can slowly release the H_2_S (*k*_off_ = 0.053 s^–1^).^60^ In the presence of O_2_, however, most H_2_S is rapidly oxidized to thiosulfate and iron-bound polysulfides through poorly characterized intermediates (eqs 13–15).^60,63^ Iron-bound polysulfides can further react with endogenous GSH to yield glutathione persulfide, which is more reactive than GSH and has been shown to be an important inhibitor of lipid peroxidation.^63−65^
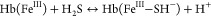
12
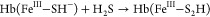
13
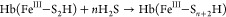
14
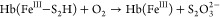
15H_2_S can also react with Hb to generate the green sulfhemoglobin. The most frequent formation of sulfhemoglobin in blood has been associated with misuse of sulfadrugs, rather than H_2_S poisoning.^66^ This green sulfhemoglobin results from the irreversible covalent reaction of sulfur to the pyrrole ring and leads to a 135-fold decrease in oxygen affinity.^67^ The mechanism of sulfhemoglobin formation is not clear, but it is postulated to involve reaction of H_2_S with an oxoferryl (Hb(Fe^IV^=O) Por^•+^ or Hb(Fe^IV^=O)) intermediate.^68^ The reactions of H_2_S with Hb are likely important in regulating the biological actions of H_2_S in the vascular system.

## 4 Antioxidant Systems in RBCs

The RBC contains several antioxidant systems that allow it to cope with extensive oxidative stress. These include low molecular weight systems involved in cytosolic protein and membrane lipid protection and enzymatic systems that can react and reduce mostly water-soluble oxidants. The following section gives a detailed description of the most relevant antioxidant systems in RBCs.

**Superoxide dismutases** (SODs, EC 1.15.1.1) are enzymes that catalyze the dismutation or disproportionation of two molecules of O_2_^•–^ to O_2_ and H_2_O_2_ (eq 2).^69^ Interestingly, this reaction occurs spontaneously (*k* = 2 × 10^5^ M^–1^ s^–1^), yet it is accelerated 4 orders of magnitude (2 × 10^9^ M^–1^ s^–1^) by these enzymes. Considering the relatively high abundance of SOD and diffusion-limited reactivity, the enzyme-catalyzed reaction is the principal O_2_^•–^ dismutation mechanism in aerobic organisms.^70,71^

SODs are metalloenzymes classified by the metal ions they bind. There are three forms in mammals: SOD1 and SOD3 that are copper−zinc superoxide dismutases (Cu/Zn SOD), and SOD2 that is a manganese SOD (MnSOD).^71,72^ RBCs contain Cu/Zn SOD1 which is accountable for at least 95% of the Cu content, and it is present at an approximate concentration of 4 μM.^73,74^ SOD1 is a homodimeric protein of 32 kDa in which each monomer contains a Cu^II^ and a Zn^II^ ion in its structure.^75^ The catalytic metal is Cu^II^, while Zn^II^ has a rather indirect role in catalysis since it stabilizes the active site structure and adjusts its redox potential.^76^ During catalysis, the Cu^II^ ion is reversibly reduced and oxidized by two consecutive encounters with O_2_^•–^ (eqs 16 and 17).
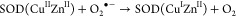
16
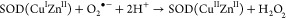
17In the resting state, the oxidized Cu^II^ binds the oxygen atom of a water molecule and is connected to the Zn^II^ ion by a histidine residue (His63). The first O_2_^•–^ travels down a positively charged active site channel to bind the Cu^II^ and displaces the water molecule. Then, the bound O_2_^•–^ reduces Cu^II^ to Cu^I^ which leads to O_2_ formation, the loss of the Cu–His61 coordination bond, and His63 protonation (eq 16). The second O_2_^•–^ binds to the Cu^I^ and oxidizes it to Cu^II^, while it receives two protons coming from the His63 and the solvent, which yield H_2_O_2_ (eq 17). As the H_2_O_2_ leaves the active site, the Cu–His63 coordination bond is reestablished, and the enzyme returns to the resting state.^77,78^

SOD1 activity is correlated with RBC physiology, protecting RBC proteins against O_2_^•–^-mediated damage. Studies on mice RBCs lacking SOD1 have shown an early increase in their size and a decreased life span (60% of that of control mice), ultimately leading to anemia. Furthermore, these RBCs exhibited high oxidant and metHb levels that had a concomitant increase with age. Moreover, SOD1-deficient RBCs exhibited bound immunoglobulins, and deposits of immune complexes were found in the glomeruli of these mice, which is a hallmark of autoimmune pathology.^79^ All these effects have been proven to be significantly reverted by the transgenic expression of SOD1.^80^ RBCs from SOD1 knockout mice also exhibited high levels of oxidized carbonic anhydrase 2 (CA2), which leads to its proteasomal degradation and RBC malfunction. Moreover, the accumulation of oxidized CA2 alters proteasomal activity and disturbs protein homeostasis in the RBC.^81^

Additionally, in human RBCs, SOD1 can be post-translationally phosphorylated and/or glutathionylated near the dimer interface. Specifically, the glutathionylation of Cys111 disrupts the dimer interface and yields SOD1 monomers that are significantly less active.^82^

Overall, SOD1 plays a main role in the RBC antioxidant system because it scavenges the O_2_^•–^ that is continuously produced by oxyHb autoxidation. Although SOD1 activity prevents generation of more potent oxidants such as peroxynitrite (Figure 1), its reaction yields H_2_O_2_, which can further oxidize cellular components and/or produce HO^•^. Therefore, SOD1 must act concertedly with the H_2_O_2_ reduction systems in order to complete its antioxidant function.

**Peroxiredoxins** (Prx, EC 1.11.1.15) are peroxidases that reduce H_2_O_2_ and other hydroperoxides (ROOH), using highly reactive cysteine residues. They are ubiquitous, abundant, present in all cell compartments, and indispensable for aerobic life. There are three Prx isoforms in RBCs, namely, Prx1, Prx2, and Prx6.^83^ Among them, Prx2 is the most abundant in the RBC with a concentration of 240–410 μM.^9,84,85^

**Figure 2:**
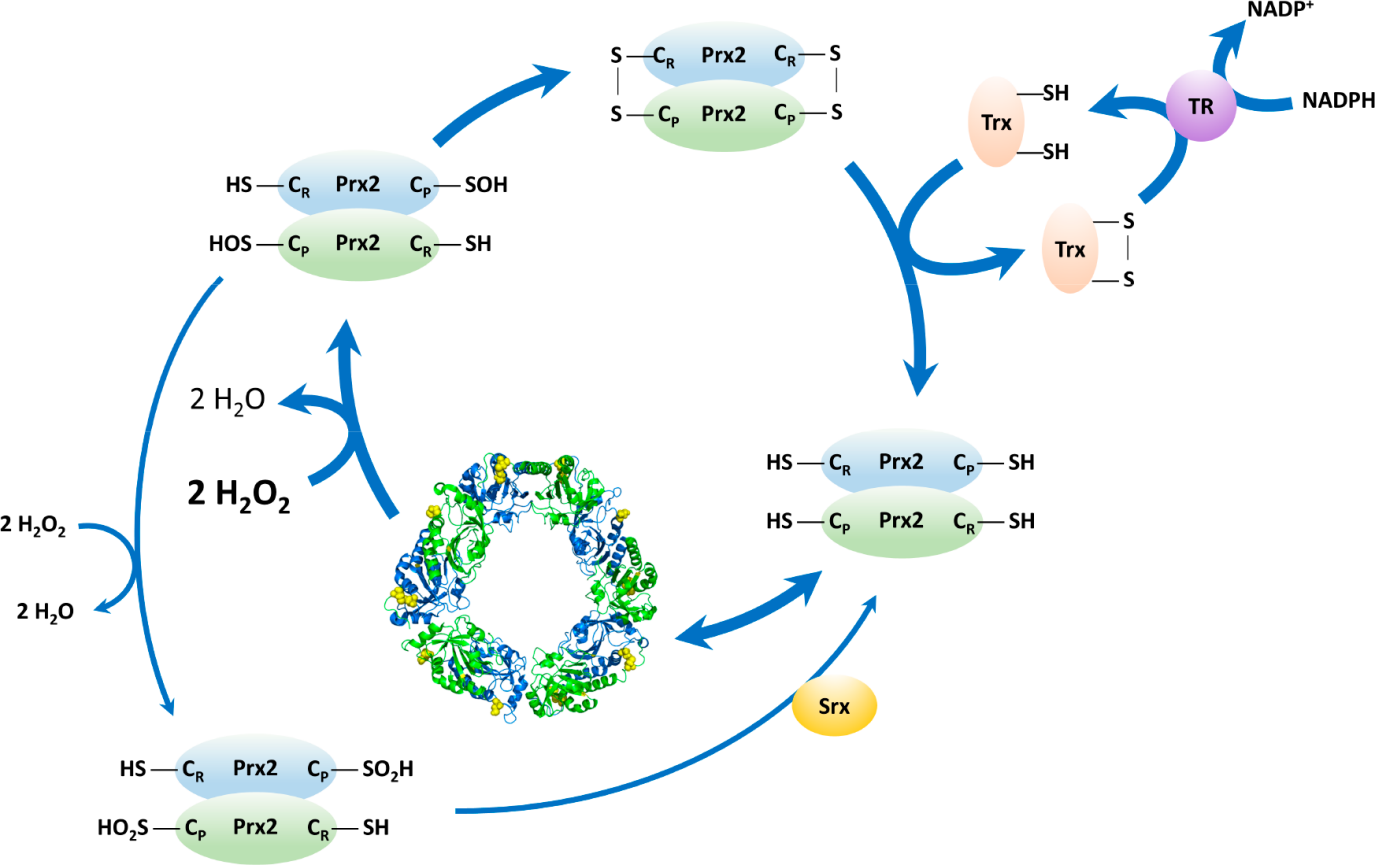
Peroxiredoxin activity. The reduced Cys52 in Prx2 (C_P_SH) is oxidized by H_2_O_2_ and other oxidants to sulfenic acid (C_P_-SOH). This C_P_SOH reacts with the C_R_SH, forming an intermolecular disulfide bridge. The disulfide-oxidized Prx2 is predominantly a dimer and is reduced by Trx, TR, and NADPH. The oxidized C_P_SOH can alternatively react with a second oxidant molecule to yield the hyperoxidized sulfinic acid (C_P_SO_2_). The latter can either be repaired to the active enzyme by sulfiredoxin (Srx) or form stacked decamer high molecular weight structures. The structure of decameric Prx2 (5IJT) shows reactive cysteine residues in yellow, and each dimer is shown in green and blue, as sides of the pentagon.

Prx2 belongs to the Prx1 class of the Prx family, also known as typical two-cysteine Prx. These Prx are homodimers (44 kDa) arranged in a head-to-tail fashion that further form toroid-shape homodecamers (220 kDa) (Figure 2). Dimerization is necessary for complete active site folding since each monomer has two halves of an active site: the amino-terminal (N-ter) head and the carboxi-terminal (C-ter) tail. During catalysis, the N-ter active site peroxidatic cysteine (C_P_) reacts with H_2_O_2_ to form a sulfenic acid on C_P_ (C_P_SOH) (Figure 2). Then, C_P_SOH reacts with the C-term active site, resolving cysteine (C_R_) from an adjacent subunit (Figure 2), to form an intermolecular disulfide bond that is later reduced by the thioredoxin/thioredoxin reductase (Trx/TR) system at the expense of NADPH. Occasionally, the C_P_SOH can react further with H_2_O_2_ to form sulfinic (C_P_SO_2_) or sulfonic acid (C_P_SO_3_), which reversibly or irreversibly inactivates the enzyme.^86^

The Prx2 C_P_ can react with both H_2_O_2_ and peroxynitrite extremely fast, with rate constants of 1 × 10^8^ and 1.4 × 10^7^ M^–1^ s^–1^, respectively.^45,49,88^ Nevertheless, the catalytic cycle of Prx2 is significantly delayed by the C_P-_-C_R_ disulfide formation (0.3 s^–1^), which makes the enzyme prone to hyperoxidation, leading to the accumulation of C_P_SO_2_ and/or C_P_SO_3_. Prx2 switches its oligomeric form based on the redox state of C_P_ and C_R_: the dithiol is a stable decamer, while the disulfide-bonded Prx2 is predominantly a covalent dimer.^89,90^ Prx2 in the RBCs is present mostly in the reduced state, and harsh oxidative insults are needed to accumulate oxidized Prx2.^83^ In some cases, the recycling of Prx2 is compromised by low NADPH availability, such as in G6PD deficiencies that will be discussed below.^91^

Prx2 is involved in different aspects of the RBC physiology. From an early stage, highly concentrated Prx2 is necessary in the erythropoiesis process, particularly in the erythroblast, acting as an antioxidant when Hb synthesis is at its peak and large amounts of heme and iron are handled.^92^ The absence of Prx2 at this stage has shown defective erythropoiesis and iron toxicity.

Furthermore, Prx2 specifically protects Hb against oxidation and is fundamental for the stabilization of its structure. Mice models lacking Prx2 show increased Hb oxidation, Heinz body precipitation, and hemolytic anemia,^93^ despite having intact catalase and GPx functionality.^94,95^ It has also been shown that the decameric state of Prx2 is needed to prevent H_2_O_2_-induced Hb aggregation.^96^

A small part of the RBC’s Prx2 pool is located in the cell membrane, where it has been associated with the cytoplasmic domain of a band 3 anion transport protein, spectrin, and the Gardos channel.^97−99^ Although the function of Prx2 in the RBC membrane is still elusive, the increase of membrane-bound Prx2 is a marker of RBC oxidation and stress.^100,101^ Interestingly, Prx2 also exhibits functions outside the RBC, as an enhancer of the cytotoxic activity of natural killer cells against tumor cells and as a proinflammatory cytokine that is excreted in exosomes.^102,103^

**Thioredoxin** is a small monomeric protein (12 kDa) which has a conserved active site sequence Cys-Gly-Pro-Cys. The N-terminal Cys attacks the target protein disulfide, generating a transient mixed disulfide (eq 18) that is then reduced by the C-terminal Cys in the active site of Trx to generate an intramolecular disulfide in Trx and the protein thiol (eq 19).^104^ RBCs contain Trx1 that participates in antioxidant defenses, acting as electron donors to several enzymes, including Prx2, and is reduced by Trx reductase (TR). Secreted Trx1 can mediate immune responses in association with Trx-interacting protein.^8^
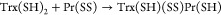
18
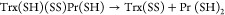
19

**Thioredoxin reductase** (TR, EC 1.8.1.9) is a homodimeric selenocysteine-containing flavoprotein member of the pyridine nucleotide-disulfide oxidoreductase family. RBCs contain TR1, and each subunit contains FAD and NADPH binding domains. The dimer is accommodated head to tail. The electrons are transferred from NADPH to FAD (eq 20), then to the N-terminal redox-active dithiol (eq 21), then to the C-terminal selenylsulfide in the other subunit (eq 22), and finally to the disulfide substrate (eq 23).^105^
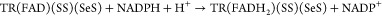
20
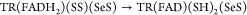
21
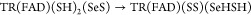
22
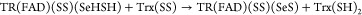
23Substrates of TR include Trx, glutaredoxins, and others.^106^ Although a lower activity of TR has been measured in human RBCs compared to other cells, it is enough to keep Prx2 in the reduced state.^83^ The TR/Trx system appears to be connected to the GR/GSH system. For instance, when TR is downregulated, Trx can be reduced by Grx/GSH, and when GR is downregulated, TR can reduce GSSG to GSH.^106^

**Catalases** (EC 1.11.1.6) are ubiquitous enzymes that catalyze the decomposition of H_2_O_2_ into water and O_2_. They can be organized into four main groups: monofunctional catalases (typical catalases), bifunctional catalase-peroxidases, nonheme catalases, and miscellaneous proteins with minor catalytic activities.^107^ Human RBC catalase is found at a concentration of 11–12 μM (subunit concentration) and belongs to the group of monofunctional catalases. It is a tetrameric enzyme consisting of four identical subunits of 59.7 kDa, and each subunit contains a heme group, iron(III) porphyrin IX, and a tightly bound NADPH molecule.^34,108^

The decomposition of H_2_O_2_ occurs in two steps. In the first step, H_2_O_2_ oxidizes the heme iron (Fe^III^) to form the intermediate compound I, a π-porphyrin cation radical containing Fe^IV^ (catalase Fe^•IV^=O) (eq 24). The rate constant for this step per subunit is *k* = 0.6 × 10^7^ M^–1^ s^–1^. One of the protons of the H_2_O_2_ molecule is transferred from one end to the other via a histidine residue in the active site, and this polarizes and breaks the O–O bond in the H_2_O_2_. In the next step (*k* = 1.7 × 10^7^ M^–1^ s^–1^, per subunit), a second H_2_O_2_ molecule acts as a reductant, producing water and O_2_ and returning the enzyme to the Fe^III^ resting state (eq 25).^109,110^ Unlike other enzymes, it is not possible to saturate catalase with H_2_O_2_, and kinetics follows a first-order reaction on H_2_O_2_ concentration.^111^
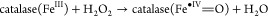
24
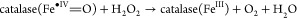
25Despite being categorized as monofunctional, typical catalase can catalyze the oxidation of two-electron donors other than H_2_O_2_ from compound I. Compound I can also be reduced to inactive compound II by one-electron donors (eq 26), and this may be reduced to the native form by another one-electron reduction (eq 27). When compound II reacts with H_2_O_2_, inactive compound III is formed (eq 28).^112,113^
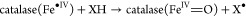
26
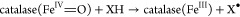
27
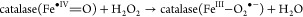
28The oxidizing and reducing power during the normal catalytic cycle of catalase comes from H_2_O_2_ (eq 24 and eq 25), so NADPH does not appear to be essential for catalytic activity. Therefore, several hypotheses have been proposed about the role of NADPH in catalase, the main one being that it protects the enzyme from inactivation by H_2_O_2_ by preventing the formation of compound II.^112,113^

**Glutathione peroxidase** (EC 1.11.1.9) is part of the thiol-dependent antioxidant systems of RBCs. It catalyzes the reduction of hydroperoxides through a process that involves GSH, glutathione reductase (GR), and NADPH. Eight different glutathione peroxidases (GPx1–GPx8) are found in mammals, with GPx1 being the predominant one in RBCs at 1.5 μM.^23,75^ GPx4 is also present, although it is 20 times less abundant.^9^ Both isoforms are selenoenzymes, with an active site consisting of a catalytic tetrad formed by a selenocysteine residue, along with a glutamine, a tryptophan, and an asparagine.^114−116^ They differ, however, in their oligomeric state and their substrates. GPx1 is a homotetramer and reacts more rapidly with H_2_O_2_ and other small organic hydroperoxides, whereas GPx4 is monomeric and reacts more rapidly with larger and more complex molecules, such as phospholipid and cholesterol hydroperoxides, even when bound to the membrane surface.^117−119^

The catalytic cycle of GPx is based on a ping-pong mechanism and can be separated in two half-reactions, an oxidative and a reductive phase.^120,121^ During the oxidative phase, the hydroperoxide is reduced upon reaction with the active site of the enzyme, while its selenocysteine residue is oxidized to selenenic acid (*k*_1_ H_2_O_2_ = 4.1 × 10^7^ M^–1^ s^–1^, *k*_1_*t*-BuOOH = 4.2 × 10^6^ M^–1^ s^–1^) (eq 29).^122,123^ In the reductive half-reaction, the enzyme is regenerated in two subsequent steps. A first molecule of GSH binds to the enzyme through a selenosulfide bond (eq 30), which is then broken by a second GSH, yielding glutathione disulfide (GSSG) and the reduced form of the enzyme (eq 31).^122,124,125^
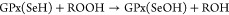
29
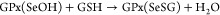
30
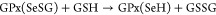
31While GPx1 has a high specificity for GSH, this is not the case for all the GPx isoforms, as a gradual loss in substrate specificity has been observed. GPx4 can accept other protein thiols and even thiol groups in its own structure as electron donors.^117,126^

RBCs from GPx1 knockout mice showed increased cell susceptibility to lysis by organic peroxides, such as *t*-butyl hydroperoxide and cumene hydroperoxide.^127,128^ In these cases, the oxidative damage was often observed at the membrane level, with the appearance of newly oxidized thiols.^129^ It was also reported that GPx1 can translocate to the membrane of RBCs in conditions of oxidative stress, before Prx2 or catalase.^130^ In addition, a recent report indicates the anticorrelation of RBC hemolysis with functional Gpx4 (lyso-phospholipids).^131^ These results support the theory that, in RBCs, the main role of GPxs is to protect the lipid membrane from oxidative attack by hydroperoxides of a different nature.

**Glutathione reductase** (GR, EC 1.6.4.2) is a flavoenzyme that catalyzes the recycling of GSSG back to GSH at the expense of NADPH. With a homodimeric structure, both GR subunits are connected via a disulfide bond. Each one can be divided into four domains and present NADPH, FAD, and GSSG binding sites. The FAD domain holds a redox-active disulfide that takes part in the reduction of GSSG.^132^

GR presents a ping-pong mechanism of catalysis, with a cycle that can be separated in two, oxidative and reductive, half-reactions.^121^ In the beginning, NADPH binds to the enzyme and reduces the flavin (eq 32), which in turn establishes a charge-transfer complex with one of the cysteines of the active site (Cys63), breaking the previous disulfide bond (eq 33). NADP^+^ is released and replaced by a new molecule of NADPH. Next, a GSSG forms a mixed disulfide with the enzyme (eq 34), and after its reduction, two molecules of GSH are produced, along with the regeneration of the oxidized enzyme (eq 35).^133^
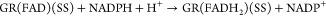
32
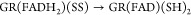
33
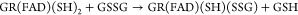
34
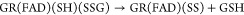
35In RBCs, GR is found predominantly in its reduced form since the concentration of NADPH is five times larger than the Km for the enzyme.^134^ This allows GR to continuously maintain the levels of GSH in the millimolar range as well as preserve the balance of NADPH and NADP^+^ pools in the pentose phosphate pathway.^135^

**Glutaredoxins** (Grx, EC 1.20.4.1) are ubiquitous cysteine-dependent enzymes that catalyze both the formation and reduction of mixed disulfides between protein thiols and GSH.^136^ They can be classified into two groups: dithiolic and monothiolic. The first group are two-cysteine oxidoreductases that get their reduction equivalents from either GSH or TR. On the other hand, monothiolic Grx lacks oxidoreductase activity and has only one active site cysteine that it uses alongside GSH to assemble and transfer iron–sulfur clusters ([Fe–S]) to proteins.^137,138^

Two Grxs are found in the mature RBC, Grx1 and Grx3, and their concentration range is 4–8 μM and 0.6–0.8 μM, respectively.^9^ Grx1 is a two-cysteine Grx that can reduce both protein disulfides and protein–GSH mixed disulfides (deglutathionylation). In the first case, the amino-terminal (N-ter) active site cysteine attacks the protein disulfide to form a mixed disulfide between Grx and the protein (eq 36). Then, the C-terminal cysteine forms an intramolecular disulfide bond with the N-ter cysteine which yields the reduced protein (eq 37). Later, the oxidized Grx is reduced by two molecules of GSH to restore the dithiolic enzyme and form GSSG (eq 38), which ultimately will be reduced to GSH at the expense of GR and NADPH.
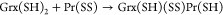
36
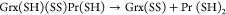
37
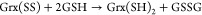
38In the case of deglutathionylation, the enzyme exhibits classical ping-pong catalysis, where first the N-ter cysteine reacts with the glutathionylated protein or low molecular weight thiol, yielding glutathionylated Grx and reduced protein. Then, GSH reduces the Grx–GSH mixed disulfide, to form reduced Grx and GSSG, which is later reduced by GR and NADPH.^139,140^

Inside the RBCs, Grx1 has been found responsible for the deglutathionylation of Hb, phosphofructokinase, and the reduction of low molecular weight disulfides.^141^ Glutathionylation of Cys93 of the Hb β-chain is thought to be a protection mechanism against oxidation, yet it can disrupt the interaction between α and β subunits, affecting O_2_ and heme binding. Additionally, it has been shown that Grx1 reduces pyruvate kinase and restores thiol groups of proteins in the RBC membrane.^142^ Grx1 is also responsible for the reduction of dehydroascorbate to ascorbate (vitamin C), which acts as a reducing agent within RBCs.^143^

On a different note, Grx3 is a cytosolic, monothiolic, multidomain Grx, whose role in the mature RBC is still elusive. Nevertheless, studies on a zebrafish model propose that the function of Grx3 is important at the early erythroblast stage, specifically in the maturation of [Fe–S]-containing proteins, the heme synthesis pathway, and iron uptake and distribution.^144^

**Figure 3:**
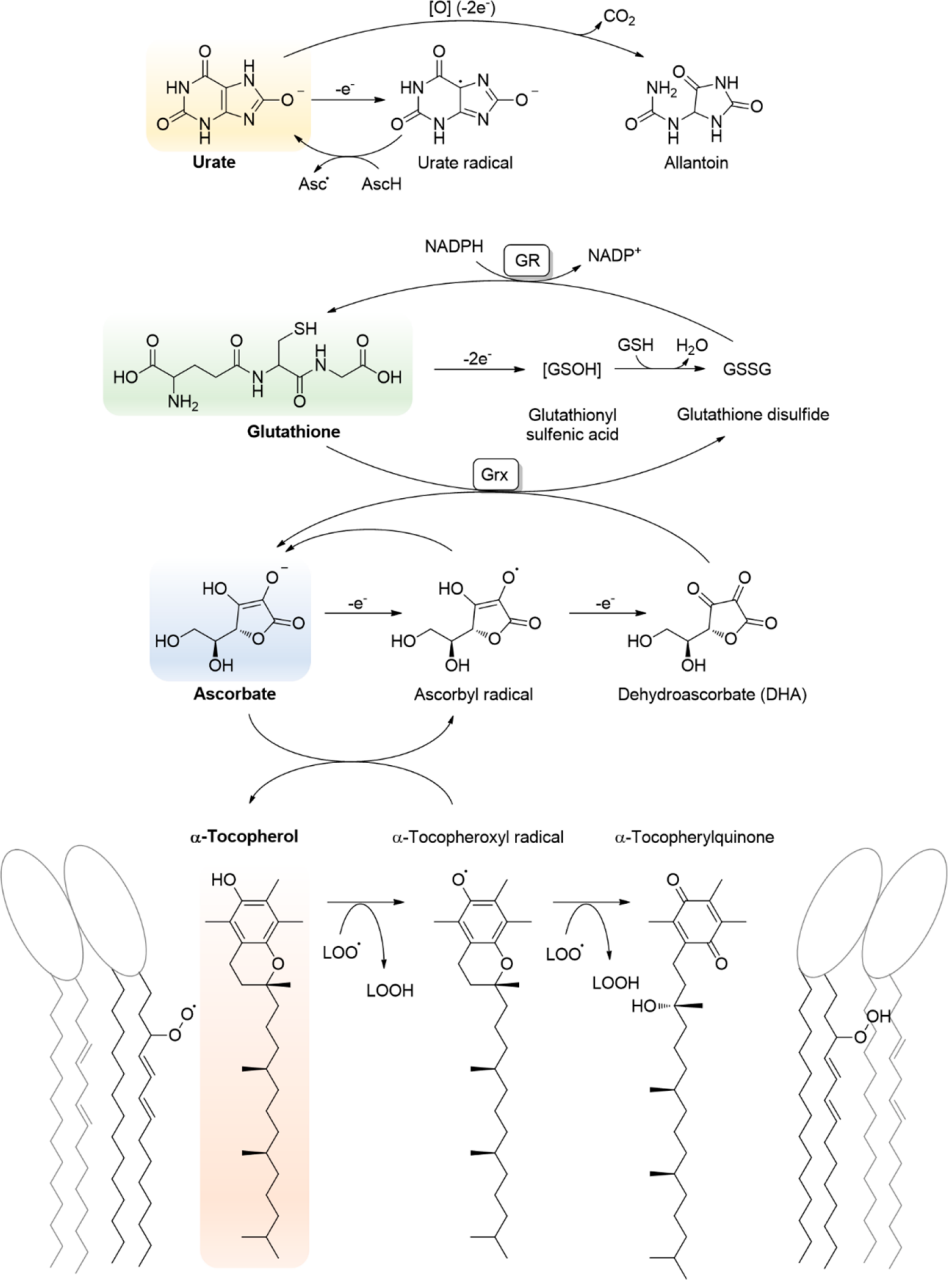
Main low molecular weight antioxidants in RBCs are the water-soluble urate, glutathione, and ascorbate and the lipid-soluble α-tocopherol. These antioxidants can form stable radicals after one-electron oxidation that can be repaired by other antioxidants. The ultimate source of reducing power is given by NADPH, which can be used to yield GSH, which can be used to reduce DHA to ascorbate, which can reduce the urate radical and also the α-tocopheroxyl radical back to their reduced forms.

**Glutathione** (GSH), the tripeptide γ-glutamyl cysteinyl glycine (Figure 3), is considered the main intracellular low molecular weight antioxidant but is also a key determinant of redox signaling and xenobiotic metabolism and one of the most important ways of reducing power in the cell.^145^ In RBCs, the intracellular concentration of GSH is relatively high (0.4–3 mM^146,147^), and the normal physiological GSH/GSSG ratio is higher than ten.^146^ Biosynthesis of GSH occurs in the cytosol in a tightly regulated manner. Key determinants of GSH synthesis are the availability of the sulfur amino acid precursor, cysteine, and the activity of the rate-limiting enzyme, glutamate cysteine ligase (GCL, EC 6.3.2.2). GCL is a heterodimer composed of a catalytic (GCLC) and a modifier (GCLM) subunit.^148^ The holoenzyme is regulated by reversible protein phosphorylation and pyridine dinucleotide phosphate-dependent allostery.^149^ Glutathione synthetase (GS, EC 6.3.2.3), the second enzyme in GSH synthesis, catalyzes the condensation of γ-glutamylcysteine and glycine, to form GSH.^146^ Both enzymes depend on ATP production by glycolysis in the RBC.^150^

Cysteine for GSH synthesis is taken up from plasma by facilitated diffusion (L-transport system) and secondary active transport (alanine-serine-cysteine transporter, Asc-1) by RBCs.^151,152^ The liver and kidney play a fundamental role, providing GSH as a cysteine precursor for interorgan exchange.^153^ Hepatic GSH is transported into blood and rapidly degraded to cysteine in the circulation by plasma-membrane-bound enzymes γ-glutamyltranspeptidase (γ-GT) and dipeptidases of the kidney and other organs.^154,155^ γ-Glutamyl-cycle-encompassing GSH synthesis, transport, and catabolism coordinate the redox state of cells and tissues and thiol homeostasis.^156^ Transsulfuration and thiol/disulfide exchange reactions are additional sources of plasma cysteine.^157,158^

A significant percentage of GSH is produced de novo daily by RBCs to compensate the active export of GSSG and GSH conjugates.^159,160^ Carbon monoxide poisoning as well as oxidants like peroxynitrite promote the release of both GSH and GSSG from RBCs.^160,161^ The multidrug resistance-associated proteins (Mrp/Abcc) mediate GSH export and homeostasis in a variety of cells.^162,163^ Mrp/Abcc-1, -4, -5, and-10 were identified by mass spectrometry in RBC membranes.^9^ The Mrp proteins, in addition to mediating GSH efflux, also export GSSG, *S*-nitrosoglutathione, GSH–metal complexes, as well as other GSH *S*-conjugates. The ability to export both GSH and oxidized derivatives of GSH provides these transporters with the capacity to directly regulate the cellular thiol-redox status and, therefore, the ability to influence signaling and biochemical pathways.^164^ As mentioned above, GSH is regenerated from GSSG by the NADPH-dependent GR (Figure 3).

**Ascorbate** (vitamin C, AscH) cannot be synthesized by humans and must be obtained from the diet. At neutral pH, most of it is present in the anionic ascorbate form rather than as ascorbic acid. Ascorbate is an electron donor that can be oxidized by either one electron to the ascorbyl radical or two electrons to dehydroascorbate (DHA), but this is usually rapidly reduced back to ascorbate (Figure 3).^165^ Ascorbate can react with free radicals in the cytosol and also keep α-tocopherol (vitamin E) in the reduced state in the plasma membrane (Figure 3).^166−168^

Unlike other human cells, there is no active transport for ascorbate in RBCs.^165^ Instead, DHA is transported into the RBC by glucose transporters GluT, where it is reduced back to ascorbate by GSH and Grx.^169^ The concentration of DHA in plasma is estimated to be 1–2% that of ascorbate.^165^ The concentration of ascorbate in human RBCs is directly proportional and slightly lower than in plasma, amounting to 35 μM in average.^170^ At this concentration, ascorbate will likely not act as a direct free radical scavenger but may aid in keeping α-tocopherol reduced in the membrane and participate in enzymatic reactions. Human RBCs contain the duodenal cytochrome b561 isoform in the membrane that was shown to transport reducing equivalents from ascorbate in the cytosol to the exterior and may contribute to maintaining plasma ascorbate in the reduced state.^171^

RBCs from type 2 diabetes patients are mechanically more fragile than control RBCs.^172^ It was proposed that excess glucose competes with DHA transport and leads to lower intracellular concentration of ascorbate that then leads to increased membrane rigidity and cell fragility.^173^ The causes are not clear and may involve enzymatic reactions that use ascorbate as a substrate rather than antioxidant effects or a combination of both.^173^

The addition of either ascorbate or DHA to blood stored for transfusion only slightly decreased storage lesions.^174,175^ In contrast, the addition of ascorbate together with *N*-acetyl cysteine (NAC) to RBCs stored for transfusion resulted in overall improvement of RBC quality, in particular, GSH and α-tocopherol levels, leading to lower rates of lipid oxidation. The glycolytic flux was diminished, but ATP and NADH were higher than in the control, and NADPH increased only transiently. A decrease in hemolysis was observed only for 21 days of storage.^176^ Millimolar concentrations of ascorbate obtained by high dose intravenous infusion of vitamin C were found to increase metHb formation and Prx2 oxidation, suggesting that high concentrations may be detrimental to RBCs.^177^

**Vitamin E** encompasses eight fat-soluble compounds containing a chromane ring with a hydroxyl group at C-6 and a polyprenoid side chain, with three isopentyl units at the C-2 position. When the polyprenoid chain is saturated, the isomers are tocopherols, and when it is unsaturated, they are tocotrienols. According to the number and position of methyl groups in the aromatic ring, they are called α, β, δ, and γ tocopherol. These compounds are synthesized mainly by plants and cyanobacteria. Although γ-tocopherol is the most abundant in the diet, α-tocopherol shows greater bioavailability in plasma and human tissues (Figure 3).^178,179^ It is preferentially retained by the organism, thanks to the α-tocopherol transfer protein, which is expressed in the liver and presents greater selectivity for α-tocopherol than other compounds.^180,181^

Although there is some controversy about its main role, α-tocopherol is considered the most important antioxidant-protecting membrane lipid against oxidative damage.^182,183^ Lipid peroxidation mainly affects polyunsaturated fatty acids (PUFAs) and leads to the formation of lipid hydroperoxides, lipid alcohols, and aldehydes. Membrane lipid peroxidation begins with the attack of reactive species capable of removing a hydrogen atom from a PUFA. PUFAs are particularly susceptible to these oxidants since they possess easily oxidizable bisallylic hydrogens.^184^ Hydrogen abstraction leads to a carbon-centered radical that tends to stabilize by molecular rearrangement to produce a conjugated diene, which rapidly reacts with oxygen to give a lipoperoxyl radical. Lipoperoxyl radicals abstract hydrogen atoms from other lipid molecules, forming a lipid hydroperoxide and fueling the chain reaction of lipid peroxidation.^185^ The hydroxyl group in α-tocopherol competes with the unsaturated chains for the reaction with the lipoperoxyl radical, forming a less reactive tocopheroxyl radical, thus preventing the propagation of the chain reaction (Figure 3).^35^ This radical can be reduced to α-tocopherol by ascorbate through its oxidation to ascorbyl radical, two of which can dismutate to ascorbate and DHA.^168,186^ Alternatively, the tocopheroxyl radical can be further oxidized to the stable form, α-tocopherylquinone (Figure 3).

The concentration of α-tocopherol in RBCs has been determined to be 1.7–7.8 μM.^187−193^ This concentration is in agreement with kinetic predictions that indicate that the α-tocopherol/lipid ratio in membranes must be of the order of 1/1000 in order to be an effective chain-breaking antioxidant.^35^

Interestingly, it has been seen that intracellular parasites, including the RBC-infecting *Plasmodium falciparum*, are capable of synthesizing tocopherol as a defense mechanism against oxidative stress.^194−197^

**Uric acid** is the final product of the catabolism of purine bases. Its formation is catalyzed by the enzyme xanthine oxidase (XO, EC 1.17.3.2), which converts hypoxanthine to xanthine and xanthine to uric acid. This series of reactions uses O_2_ as an electron acceptor, thus yielding O_2_^•–^ and H_2_O_2_ as secondary products. The antioxidant potential of uric acid resides in its ability to react with strong oxidants, such as peroxyl radicals and HO^•^. Urate can be oxidized by one-electron transfer to a urate radical anion, which can then be recycled in the presence of ascorbate (Figure 3). In a two-electron transfer reaction, urate is oxidized predominantly to allantoin (Figure 3) and also parabanic acid, oxaluric acid, and other compounds.^198−200^

In mammals, xanthine oxidase is mostly located in the liver, but it has also been found in epithelial cells of mammary glands and capillary endothelial cells in adipose, cardiac, and lung tissues.^201^ The RBC, on the other hand, does not show xanthine oxidase activity, so uric acid is not expected to be produced by the RBC.^202^ However, they are exposed to high concentrations of uric acid present in plasma (200–400 μM), where it represents one of the most important soluble antioxidants, accounting for 30–65% of the peroxyl-radical-scavenging capacity.^203,204^ Furthermore, it was revealed in earlier studies that RBCs are able to uptake uric acid, but it should be noted that there is not a consensus value reported for the intracellular concentration reached, and the mechanism is not fully understood. The main route was proposed to be an active transporter, with simple diffusion occurring in the absence of ATP.^205−208^

Regarding its possible antioxidant role, in vitro studies have shown that uric acid could help neutralize the oxidant species produced from Hb autoxidation. It was reported that it can limit the rate of formation of metHb, probably by reacting with intermediates like NO_2_^•^ and ferrylHb.^205,206^ Experiments performed in RBCs from volunteers before and after physical exercise, where oxidative stress is increased, support this idea of uric acid as an antioxidant. The results exhibited an increase in uric acid concentrations inside RBCs, which decreased after an hour, following the appearance of allantoin.^209^ Other studies carried out in RBC ghosts have shown that uric acid added externally could protect RBC membranes from oxidative stress. Lipid peroxidation was observed to be diminished in ghosts exposed to *t*-butyl hydroperoxide or 2,2′-azobis(2-amidinopropane) dihydrochloride (AAPH) when treated with uric acid.^205,210,211^ Along the same lines, more recent observations made in RBCs stored for transfusion indicate there could be a beneficial effect in supplementing samples with uric acid.^212^ RBC concentrates that proceeded from donors with higher levels of uric acid in plasma manifested less deterioration during storage. Mainly, a lower percentage of echinocytes, band 3 proteolysis, and a diminished binding of calpain and Prx2 to the membrane were observed.^213^

## 5 Glucose Metabolism in RBCs and Generation of NADPH

RBCs do not contain mitochondria, and the glucose metabolism is dominated by glycolysis and the pentose phosphate pathway (PPP). Glycolysis provides ATP needed to maintain the ion gradients across the membrane and NADH for metHb reduction to oxyHb and pyruvate reduction to lactate. The PPP provides NADPH, one of the most important biological carriers of reducing equivalents, working usually as a coenzyme. This molecule is ubiquitously distributed in different cells, where it plays an essential role in redox homeostasis. In human RBCs, the concentration of NADPH has been reported to range from 16 to 44.9 μM.^214−219^

In RBCs, NADPH provides reducing power which is used by different antioxidant enzymes to fight against oxidative damage,^220−223^ and it is mainly produced by glucose-6-phosphate dehydrogenase (G6PD) and 6-phosphogluconate dehydrogenase (6PGD), enzymes involved in the PPP. Glucose-6-phosphate dehydrogenase catalyzes the first step of the PPP, converting glucose-6-phosphate to 6-phosphogluconolactone, using NADP^+^ as the substrate and Mg^2+^ as the cofactor and producing NADPH. The *K*_m_ for NADP^+^ has been reported to be 4.2 × 10^–6^ M at pH = 7.6.^224^ The third step of the PPP is catalyzed by 6-phosphogluconate dehydrogenase. This enzyme uses Mn^2+^ as a cofactor and NADP^+^ as a substrate to catalyze the decarboxylation of 6-phosphogluconate, producing ribulose-5-phosphate, CO_2_, and NADPH. A *K*_m_ = 20 μM at pH = 8 has been reported.^225^

The fluctuations of O_2_ concentrations to which RBCs are subjected in the circulation regulates the flux of glucose to generate ATP by glycolysis or NADPH by the PPP.^226^ In the pulmonary capillaries, where O_2_ concentration is relatively high, the glycolytic enzymes are inhibited by their association to band 3, pushing glucose and the flux of electrons to the PPP. Conversely, in peripheral vascular beds with lower O_2_ tensions, deoxygenated Hb binds band 3, displacing the glycolytic enzymes which became active.^227,228^ Oxidative modification of GAPDH reroutes glycolysis to PPP to fuel NADPH biosynthesis.^229,230^

NADPH is used in RBCs as a substrate of two enzymes that are relevant to support the antioxidant defenses, GR and TR. On one hand, GR recycles GSSG to GSH, which is used by GPx and Grx. On the other hand, TR keeps Trx in the reduced state, which serves as an electron delivery system that is used by Prx2 and reduction of other cysteines due to its disulfide reductase activity.^231^ As will be discussed below, glucose-6-phosphate dehydrogenase deficiency is a common human enzymopathy that leads to several phenotypes ranging from neonatal jaundice to acute anemia, indicating how important NADPH is to prevent oxidative stress in RBCs.^232^

Recent studies have shown that the exposure of RBCs to different exogenous substances (exposome), including prescribed and over-the-counter drugs, smoking, as well as drinking coffee and taurine-rich beverages, can influence glucose use and NADPH production, impacting the capacity of the cells to cope with oxidants.^220−223^

## 6 Integrating the Oxidant Challenges and the Antioxidant Defenses of RBCs

RBCs are exposed to both endogenous and exogenous oxidants. In circulation and mainly in microcirculation, RBCs come in contact with oxidants generated by surrounding cells and tissues. Endothelial cells, and leukocytes in particular, express the enzymes devoted to the production of NO^•^, O_2_^•–^, and H_2_O_2_. In leukocytes, the inducible isoform of the NOS (NOS2, EC 1.14.13.39) and the NADPH-oxidase (Nox2, EC 1.6.3.1) generates NO^•^ and O_2_^•–^.^233,234^ Both leukocytic enzymes are activated during inflammation, and in many cases MPO is released and produces HOCl. In blood-vessel-lining endothelial cells, NO^•^ and H_2_O_2_ are produced by NOS3 and Nox1, 2, 4, and 5, contributing to maintain the normal blood flow.^235^ The imbalance in the relative production of endothelial-derived relaxation and contraction factors alters endothelial physiology and may lead to impairment of the normal blood flow.^17^ Moreover, uncontrolled production of oxidants has been involved in the development of chronic diseases, such as atherosclerosis and neurodegenerative diseases.^236^ As mentioned above, NO^•^ and O_2_^•–^ are weak oxidants, but they can give rise to more potent oxidant molecules like peroxynitrite, H_2_O_2_, and HO^•^ (Figure 1).^11,237,238^

The oxidants generated both in the vasculature and by the surrounding tissues converge toward the blood. Due to their abundance and the high membrane permeability, RBCs are preferential targets for the oxidants reaching the bloodstream. In fact, uncharged NO^•^, H_2_O_2_, ONOOH, and HOCl freely diffuse through the lipid bilayer, while anion channels, like the exceedingly abundant band 3 anion transport protein, allows the permeation of O_2_^•–^ and ONOO^–^ through the RBC membrane.^26,41,239^ Once inside the RBC, the oxidants are rapidly decomposed by a complex and concerted antioxidant machinery, resulting in a marked concentration gradient across the membrane and promoting the entry and decomposition of more oxidant molecules, supporting the role of RBCs as an efficient sink of oxidants, protecting cells and tissues from uncontrolled oxidative stress.

The antioxidant systems in RBCs act in concert to detoxify most of the reactive species produced by RBCs and their surroundings. OxyHb autoxidation generates O_2_^•–^ that is rapidly converted to H_2_O_2_ by SOD1. There has been a long debate about the relative importance of the different antioxidant systems in RBCs responsible for H_2_O_2_ detoxification. Kinetics and further experimental confirmation indicate that the first line of defense of RBCs against H_2_O_2_ is Prx2, supported by its abundance and very high reaction rate (Table 1).^34^ Prx2 activity is mainly sustained by Trx/TR and ultimately NADPH from the PPP.^34^ When NADPH is depleted and Prx2 is completely oxidized, catalase acts as the second line of defense. In many experimental conditions Prx2 is completely oxidized, and catalase has been observed as the main antioxidant enzyme, resulting in a persistent confounding factor in the literature.^34^ GPx1 is expected to play a minor role in H_2_O_2_ detoxification mainly because of its lower abundance^34,85,98^ but seems to be more important in the reduction of lipid hydroperoxides.^124^

**Table 1:** Concentrations and Rate Constants of the RBC Components Responsible for H_2_O_2_ Removal

Reactant with H_2_O_2_	Concentration (μM)	Second-order rate constant (M^–1^ s^–1^)	Pseudo-first-order rate constant (s^–1^)
Peroxiredoxin-2	∼300^34,98,240^	1 × 10^8^^45,49,88^	∼30,000
Catalase	11^34^	6 × 10^6^^109^	66
Glutathione peroxidase 1	∼1^34,242^	2 × 10^7^^243^	∼20
Hemoglobin, oxyHb	20,000^244^	100^244^	2
Glutathione, GSH	1,500^246^	0.42^45^	6.3 × 10^–4^

The lipids in the membrane are protected against oxidation by α-tocopherol and ascorbate that act in concert to reduce lipid peroxyl radicals to lipid hydroperoxides. The α-tocopheroxyl radical is reduced by ascorbate, and the resulting DHA is reduced by Grx and GSH. The lipid hydroperoxides are then reduced by GPx and GSH to the corresponding alcohols to prevent the formation of the highly reactive alkoxyl radicals. Protein thiols can be protected from irreversible oxidation by glutathionylation,^245^ and this modification can be reversed by Grx1. The products of H_2_S reaction with metHb, in particular the polysulfides, could yield glutathione persulfide and other persulfides that could also protect against protein and lipid oxidation in RBCs, but this remains to be proven.

**Figure 4:**
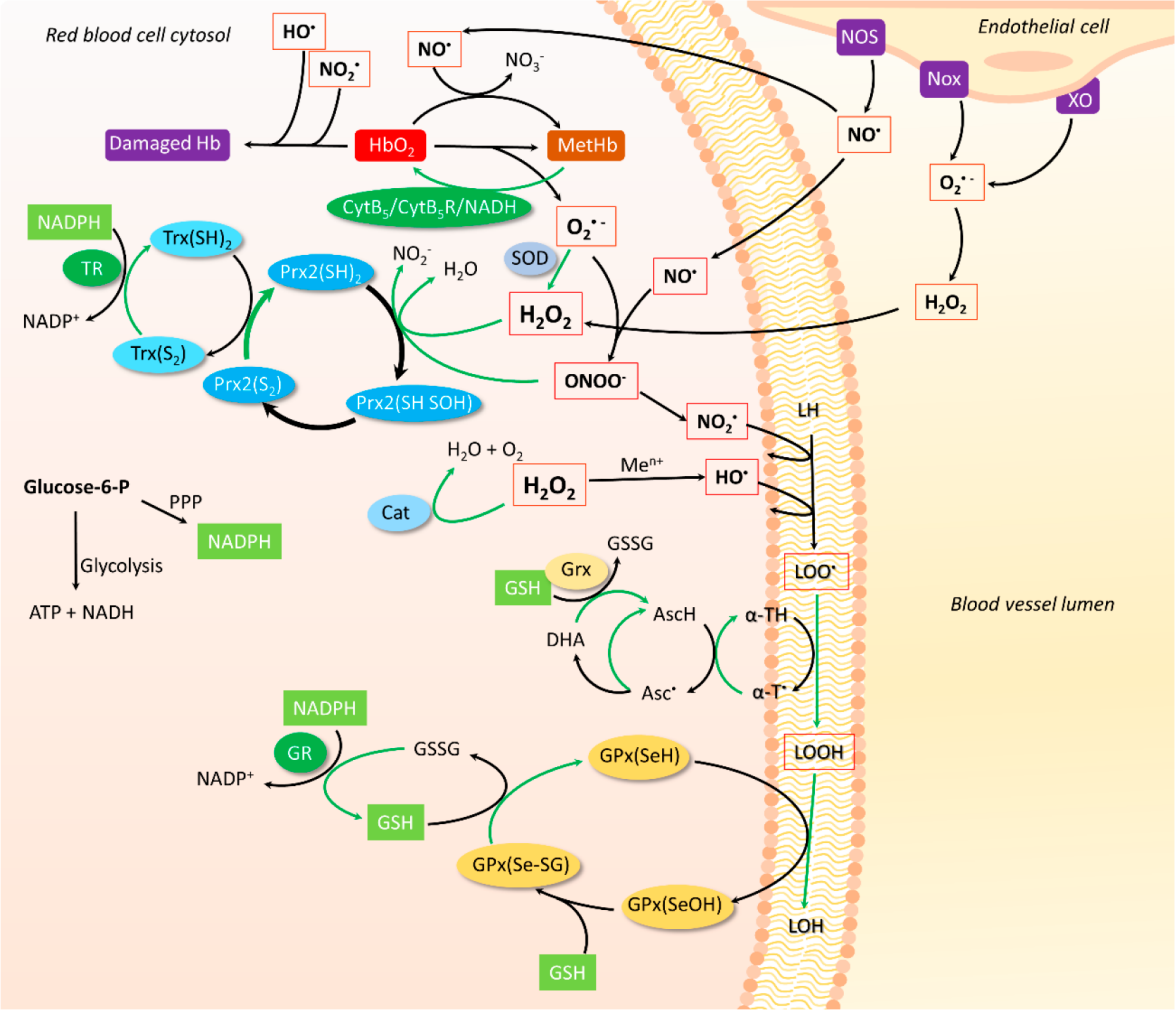
Antioxidant systems in RBCs are robust and redundant, allowing the detoxification of several oxidants (shown in red boxes) such as O_2_^•–^, H_2_O_2_, and ONOO^–^ that could lead to more potent and less selective oxidants such as NO_2_^•^ and HO^•^, which could result in hemoglobin damage, affecting RBC functionality. Oxidants can be generated endogenously or can be from other cells in the blood vessels. The RBC contains low molecular weight antioxidants, such as GSH, ascorbate, and α-tocopherol, and several enzymatic systems (in ovals of different colors). Reduction reactions are shown by green arrows. The reducing power for the antioxidant systems in RBCs is ultimately provided by NADPH from glucose-6-P and the pentose phosphate pathway. Details of the different pathways are given in the text.

Peroxynitrite will also react preferentially with Prx2, while NO_2_^•^, HO^•^, and CO_3_^•–^, which are more reactive and less selective, will react with the most abundant target, Hb. It all indicates that the RBC is well-equipped to prevent the formation of these highly reactive species that will damage Hb, essential for the main RBC function. Most of these reactions are shown in Figure 4, intended to illustrate the complexity, redundancy, and robustness of RBC antioxidant systems.

## 7 Alterations in RBC Redox Homeostasis Led to Diseases

Oxidative stress was found in several hereditary RBC diseases, including the hemoglobinopathies (sickle cell disease and thalassemia) and glucose-6-phosphate dehydrogenase deficiency. Although oxidative stress is not the primary etiology of these diseases, oxidative damage to the RBC plays a crucial role in early removal of RBCs from circulation or provoking hemolysis; thus, anemia is a common feature to all these patients. A single mutation in the β globin gene of Hb, the substitution of the sixth glutamic acid for valine (Glu6Val, HbS), is the cause of sickle cell disease (SCD). Under hypoxic conditions, the deoxygenated state of HbS polymerizes as fibers in RBCs, which deform into sickle-shaped cells that occlude capillaries and cause intravascular hemolysis. Polymerized HbS reduces cell deformability and impairs rheology and survival of RBCs. In heterozygotes, the coexistence of HbA and HbS in RBCs prevents polymerization of HbS, but SCD manifests in homozygotes. These people display anemia, feeding disorders, splenomegaly, and recurrent infections. In addition, vascular occlusion can cause cerebral infarction. There was an early report indicating HbS can autooxidize twice as fast as HbA; however, comparable rates were shown later.^246,247^ Nevertheless, hemolysis releases Hb, iron, and increased HO^•^ production via Fenton reaction, indicating oxidative stress is an important feature of SCD.^248^ Free Hb released from intravascular hemolysis of the sickle erythrocytes induces endothelial dysfunction by depleting endothelial NO^•^, heme-mediated inflammation, and iron overload.^249^ Desferoxamine as an iron chelator and a catalase mimetic were shown to decrease oxidative stress and inflammation in murine models of SCD.^250,251^ Hydroxyurea, approved by the FDA to treat SCD, increases fetal HbF with no β-chain and reduces the tendency for HbS to polymerize.^252^

Thalassemia is defined as a quantitative imbalance of α- and β-Hb chain production. α-Thalassemia develops when a gene deletion in the α-globin locus (α1 and α2) occurs. The excess of β-chains leads to the formation of Hb β4 that is stable but incapable of carrying oxygen. Conversely, β-thalassemia (or minor thalassemia) is mainly caused by a missense mutation (single amino acid substitution) in the β-globin gene. The excess of α-chains cannot form a tetramer; heme is easily released; oxidative stress in RBCs is installed; and anemia is observed. It was reported that an elevated content of GSH in RBCs significantly reduced the sensitivity of thalassemic RBCs to hemolysis and phagocytosis by macrophages.^253^

As mentioned before, glucose-6-phosphate dehydrogenase (EC 1.1.1.49, G6PD) participates in the generation of NADPH. G6PD deficiency is the most common human enzyme defect, an X-linked hereditary genetic defect due to mutations in the g6pd gene (about 140 mutations have been described, mostly single amino acid substitution). The clinical manifestation is hemolytic anemia triggered by exogenous agents like drugs and fava beans (so-called favism).^232^ The G6PD enzyme is critical to protect RBCs against oxidative stress, and subjects with deficiency in this enzyme are at risk of hemolysis under certain conditions. An increase in oxidative markers has been observed in these patients, and management of G6PD deficiency is to prevent hemolysis by avoiding oxidative stress. Successful treatment on favism-induced rats was reported with antioxidant β-carotene and also with a small-molecule activator AG1 that promotes the G6PD dimeric state on patient RBCs.^254,255^

## 8 RBC Aging in Circulation and in Packed RBC for Transfusion

Despite the effectivity of the RBC antioxidant systems, these hematic cells are constantly challenged. Moreover, in the capillaries, the increase of partially oxygenated Hb raises the rate of Hb autoxidation^256^ as well as exposure to the oxidants generated by other cells and tissues.^257^ This repeated exposure to oxidants deteriorates the capacity of RBCs to cope with them and to fulfill their physiological function. The increased metHb concentration impairs the capacity of RBCs to transport oxygen but also increases the adherence of Hb to the RBC membrane, impacting ATP synthesis and exposing its molecular components to the deleterious effects of superoxide and derived oxidants. The oxidative insult disrupts the interactions between membrane lipids and proteins and the cytoskeleton, compromising the ability of RBCs to squeeze and transverse narrow capillaries, affecting also the transport of ions and organic molecules like glucose through the membrane.^257^ The accumulation of altered proteins and lipids leads to vesiculation of the plasma membrane with the consequent release of extracellular vesicles in order to remove damaged and potentially toxic molecules.^258−260^ While vesiculation is an important homeostatic mechanism, excessive shedding of membrane parts inevitably leads to less deformable RBCs that are more prone to lysis.^261^ These aging-related changes are enhanced in RBCs affected by genetic disorders and also in RBCs exposed to oxidative stress.^260,262^

The lifespan of mature RBCs is ∼120 days. The removal of senescent or damaged RBCs involves tightly regulated molecular mechanisms, with participation of splenic and liver macrophages.^263−265^ The phagocytic response of these macrophages is triggered by the absence of CD47 and the exposure of phosphatidylserine in the outer membrane leaflet of senescent or damaged RBCs.^266^ The progressive loss of elasticity and increased rigidity are also responsible for the recognition of RBCs by the phagocytic cells.^265,267^ Another important mechanism promoting the engulfment of RBCs is triggered by the formation of antigens on the cells’ surface due to the interaction between denatured Hb and band 3, disrupting the membrane structure and leading to clustering of membrane proteins and the formation of protein aggregates. Band-3-centered protein aggregates become targets for opsonization by naturally occurring antibodies.^267−269^ Those changes also occur in some genetic disorders, such as sickle cell disease (SCD), spherocytosis, and thalassemia as well as acquired pathologies such as sepsis, malaria, or diabetes, shortening the life span of affected RBCs.^270−272^

Under storage of packed RBCs for transfusion, several aging RBC phenotypes are evident, dominated by the so-called “storage lesions”.^273^ This term brings together a set of progressive structural and metabolic changes in RBCs stored for transfusion. These lesions include the oxidation of lipids and proteins and affect RBC metabolism by compromising the activity of key enzymes, leading to a marked decrease in ATP and 2,3-diphosphoglycerate levels.^274^ The decrease in ATP deregulates cation homeostasis and alters membrane asymmetry, triggering the exposure of phosphatidylserine and phosphatidylethanolamine, normally confined to the inner bilayer.^275^ The structural disorganization of the membrane and the cytoskeleton, together with the lack of control in calcium homeostasis, leads to a progressive loss of the biconcave disc shape and the ability of the RBC to squeeze, properties that allow rapid gas exchange with the environment and traversing narrow capillary beds, characteristics of healthy RBCs.^276^

Additionally, functional and structural changes have been observed at the level of band 3.^277^ This protein, in addition to mediate the membrane Cl^–^/HCO_3_^–^ exchange, is also a key regulator of the RBC cytoskeleton dynamics and cell metabolism.^9,278^ During storage, a disruption of the interaction of band 3 with cytoskeleton proteins and a progressive clustering of the protein has been observed.^97,279^ These changes, which also involve other proteins and lipids of the membrane, lead to changes in the shape of RBCs and give rise to extracellular vesicles containing altered cellular components.^280^ Cellular reducing power is also affected as a consequence of metabolic changes, with a decrease in GSH and NADPH,^146,281^ compromising the ability of RBCs to respond to oxidative stress, both in the transfusion bag and in the circulation after transfusion. Under normal conditions, band 3 acts as a nucleation center for various enzymes of the glycolytic pathway in RBCs, regulating the flow of glucose toward ATP generation or NADP reduction, depending on the oxygen level. This fine metabolic regulation mediated by band 3 is disrupted in aged cells by oxidation, fragmentation, and nonenzymatic glycation of the protein.^278^ These structural and functional changes are reproduced in RBCs carrying altered Hb and in RBCs lacking or carrying band 3 polymorphisms.^282−285^ Moreover, altered RBCs and the extracellular vesicles released by them can trigger an inflammatory response, evolving to major complications in the transfusion recipient.^286^ Although leukoreduction has improved several parameters, concerns about the safety of RBCs stored for longer periods and the patient outcomes still persist.^287−289^ At present, most of the efforts in blood storing for transfusion are directed to decrease these aging- and oxidative-related changes in order to ensure a safe therapy for the transfusion recipients. Furthermore, there are genetic and environmental differences between donors which make blood a nonstandardized therapeutic tool. A genome-wide association study (GWAS) identified G6PD polymorphism to impair RBC recovery after transfusion and modulate disease severity in hemolytic diseases.^290−292^

## 9 Conclusions

RBCs are exposed to oxidants of endogenous and exogenous origin but are well-equipped to cope with these oxidants. The antioxidant defense includes low molecular weight molecules such as GSH, ascorbate, urate, and α-tocopherol, enzymes such as SOD1 and catalase, and multienzymatic systems, such as Prx2/Trx/TR and Gpx/GSH/GR, that ultimately depend on the reducing power provided by the PPP in the form of NADPH. Many of these enzymes react very rapidly with mildly oxidizing reactive species, such as O_2_^•–^, H_2_O_2_, and ONOO^–^, suggesting that the main purpose of these antioxidant systems is to avoid the formation of the more potent and less selective oxidants HO^•^, NO_2_^•^, and CO_3_^•–^ that will damage Hb and compromise RBC function. Even though RBCs are very robust to oxidant damage, congenital defects including hemoglobinopathies and G6PD deficiency result in less viable RBCs, related to rapid accumulation of damaged biomolecules in these RBCs. Furthermore, the RBCs that are stored for transfusion for medium to long periods of time also suffer from storage lesions related to the oxidant damage of biomolecules.

RBCs in circulation are exposed to constant oxidative stress, and maintenance of an adequate redox balance is essential to pursue their physiological function and to preserve Hb as an oxygen carrier as well as a flexible membrane for sustained microcirculation dynamics.

## Abbreviations

RBC: red blood cell
Hb: hemoglobin
GSH: glutathione
GSSG: glutathione disulfide
PE: phosphatidylethanolamine
PC: phosphatidylcholine
SM: sphingomyelin
PS: phosphatidylserine
PI: phosphatidylinositol
band 3: band 3 anion transport protein (SLC4A1)
Prx2: peroxiredoxin 2
oxyHb: oxyhemoglobin
metHb: methemoglobin
O_2_^•–^: superoxide
Nox2: NADPH oxidase 2
SOD1: superoxide dismutase 1
H_2_O_2_: hydrogen peroxide
NO^•^: nitric oxide
HO^•^: hydroxyl radical
NOS: nitric oxide synthases
ONOO^–^: peroxynitrite
ONOOH: peroxynitrous acid
NO_2_^•^: nitrogen dioxide
CO_3_^•–^: carbonate radical
MPO: myeloperoxidase
HOCl: hypochlorous acid
Trx: thioredoxin
TR: thioredoxin reductase
cat: catalase
GPx: glutathione peroxidase
GR: glutathione reductase
Grx: glutaredoxin
AscH: ascorbate
DHA: dehydroascorbate
XO: xanthine oxidase
PPP: pentose phosphate pathway
G6PD: glucose-6-phosphate dehydrogenase
6PGD: 6-phosphogluconate dehydrogenase
SCD: sickle cell disease
